# A 2D hyperspectral library of mineral reflectance, from 900 to 2500 nm

**DOI:** 10.1038/s41597-019-0261-9

**Published:** 2019-11-11

**Authors:** Laurent Fasnacht, Marie-Louise Vogt, Philippe Renard, Philip Brunner

**Affiliations:** University of Neuchâtel, Neuchâtel, Switzerland

**Keywords:** Imaging and sensing, Mineralogy

## Abstract

Mineral identification using machine learning requires a significant amount of training data. We built a library of 2D hyperspectral images of minerals. The library contains reflectance images of 130 samples, of 76 distinct minerals, with more than 3.9 million data points. In order to produce this dataset, various well-characterized mineral samples were scanned, using a SPECIM Short Wave Infrared (SWIR) camera, which captures wavelengths from 900 to 2500 nm. Minerals were selected to represent all the mineral classes and the most common mineral occurrences. For each sample, the following data are provided: (a) At least one hyperspectral image of the sample, consisting of 256 wavelengths between 900 and 2500 nm. The raw data, the high dynamic range (HDR) image, and the masked HDR image are provided for each scan (each of them in HDF5 format). (b) A text file describing the sample, providing supplementary information for the subsequent analysis (c) RGB images (JPEG files) and automated 3D reconstructions (Stanford Triangle PLY files). These data help the user to visualize and understand specific sample characteristics. 2D hyperspectral images were produced for each mineral, which consist of many different spectra with high diversity. The scans feature similar spectra than the ones in other available spectral libraries. An artificial neural network was trained to demonstrate the high quality of the dataset. This spectral library is mainly aimed at training machine learning algorithms, such as neural networks, but can be also used as validation data for other types of classification algorithms.

## Abstract (summary)

**Table:** 

Measurement(s)	mineral
Technology Type(s)	hyperspectral imagery • chemical imaging
Factor Type(s)	wavelength

Machine-accessible metadata file describing the reported data: 10.6084/m9.figshare.9963167

## Background & Summary

One of the key applications of hyperspectral imaging is classification. Various algorithms exist to determine mineralogical maps based on hyperspectral images^1^. A commonly used algorithm is the spectral angle mapper (SAM)^2^. More recently, algorithms based on Support Vector Machines^3^ have been used. Even more recently, methods based on Gaussian Process, such as GP-OAD^4^ have been employed successfully. Despite these recent advances, SAM currently remains a widely applied algorithm^5^. All of these approaches require a spectral library compiling reference spectra.

The USGS Spectral Library^6^ is commonly used for this purpose. It provides a reflectance spectrum for a wide range of minerals, usually in powder form. The USGS library was built using high quality spectrometers. This type of measurement provides a single, homogeneized measurement per sample. This type of library is designed for standard classification algorithms such as the SAM but is not ideal for machine learning algorithms.

Machine learning algorithms such as artificial neural networks became very popular. This approach requires a large amount of data per class for the training. The training dataset should be as diverse as possible, to enable the learning algorithm to generalize as much as possible. This new approach requires a new type of spectral library, which contains as much data as possible for each class.

The dataset we propose is designed to be this new type of spectral library. We use a hyperspectral camera to produce 2D images, which contains many datapoints, contrary to libraries produced by spectrometers. This diversity of the spectra measured is achieved by avoiding homogenization, by measuring mineral samples instead of powders.

Minerals were selected from the collection of the University of Neuchâtel, Switzerland, and cover all major mineral classes and of the most commonly occurring minerals^7^.

## Methods

This dataset contains scans of rock samples of the mineral collection. When ever possible, different rock samples of the same mineral were selected to ensure diversity, by having different external characteristics (such as habit or color) of the same mineral. When multiple fragments of the same rock sample were available, data acquisition was performed on each of them.

The dataset consists of three different kinds of data, each of which is described in a different subsection:

- Hyperspectral data
- 3D reconstruction and RGB images of the sample
- Text description (provides the mineral characterization).

**Fig. 1:**
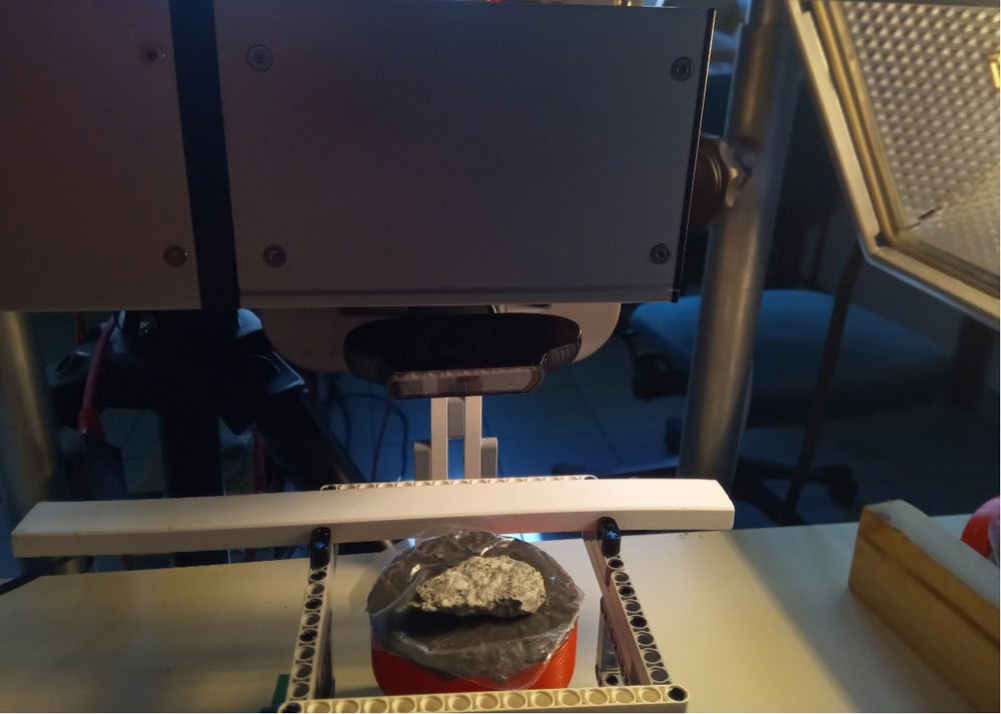
Setup to capture data. The sample is put on a support such as its upper surface is approximately horizontal. The hyperspectral camera is above the sample, there is a Logitech C920 webcam behind the sample to capture visible images for photogrammetry. Illumination is done using a halogen lamp (on the right). The white bar is the white reference (Spectralon).

The hyperspectral and photogrammetry data acquisition is entirely automatic, using the setup shown in Fig. 1. The sample is placed on a 3D-printed container (diameter: 7.2 cm) containing modelling clay, in order to have a mostly flat surface above. Thanks to the geometry of the scene, the background has a minor impact on the spectra measured. This has been verified by acquiring data twice for the same sample, but with different backgrounds.

The focus is automatically adjusted using a contrast maximization algorithm in the middle of the sample. For samples with a rough surface, the image might be slightly out of focus in some areas, leading to a decreased spatial resolution. Focus stacking algorithms were evaluated, but were finally not used as they didn’t provide a significant quality improvement. The hyperspectral camera acquires the hyperspectral data (details presented in the following section), followed by the capture of the visible images for the photogrammetry.

The spatial resolution obtained depends on the thickness of the sample, as a thick sample is closer to the camera than a thin one. In the worst case, for very flat samples, the pixel size is about 0.25 mm*0.25 mm/pixel.

Throught this section, we will often refer to data stored in files. To increase the readability, we use the following notations:

- .extension means the file ending with.extension which corresponds to the sample
- .scan.h5[/wavelengths] is the data at /wavelengths in the file ending with.scan.h5 which corresponds to the sample.

### Hyperspectral scanning and processing

The hyperspectral data acquisition was performed using a Specim SWIR hyperspectral camera. This device consists of a push-broom camera, and a mirror scanner.

The data captured should be as independant as possible of the sensor and illumination characteristics. This specific sensor returns values between 0 and 16’383 for each pixel, depending on the radiance of the sample at the specific wavelength. In practice, the value varies between a base noise level, up to a point where the sensor exhibits saturation. Between these points, the sensor value is linearly dependent on the radiance of the sample. To obtain reflectance, we need to subtract from each captured frame the base noise level (dark frame, which we obtain by capturing an image with the shutter closed), and then divide the radiance data captured from the sample by the radiance data captured on a white target (white frames). It should be noted that some pixels  are either are stuck on a given value or behave randomly. These pixels are considered as “bad pixels” and should be removed from the dataset, as the reflectance computed would be inconclusive.

Due to the geometry and the diversity of the samples, it was often not possible to find an integration time which simultaneously doesn’t overexpose some pixels, and underexposes others. Therefore, to obtain an image without saturation, a high dynamic range (HDR) approach was used, by capturing images at multiple different integration times *t*_0_, … *t*_*n*_, and then merging the images. The integration times can be seen in.scan.h5[/integration_times].

The following was captured:

1: **for** *k* ← 0 … *n* **do**          ▹ Capture a white frame for all integration time

2: Camera integration time ← *t*_*k*_

3:.scan.h5[/*white-k-d0*] ← Dark frame

4:.scan.h5[/*white-k*] ← 25 white frames

5:.scan.h5[/*white-k-d1*] ← Dark frame

6: **end for**

7:

8: **for** *k* ← 0 … *n* **do**               ▹ Capture data for all integration time

9:Camera integration time ← *t*_*k*_

10:.scan.h5[/*data-k-d0*] ← Dark frame

11:.scan.h5[/*data-k*] ← Capture data

12:.scan.h5[/*data-k-d1*] ← Dark frame

13: **end for**

Dark frame subtraction, bad pixel removal, and saturated value removal was then applied to.scan.h5[/*white-k*] (obtaining *W*_*k*_) and to.scan.h5[/*data-k*] (obtaining *D*_*k*_).

Dark frame subtraction was performed using a linear interpolation between the dark frame captured before and after the data acquisition, and was subtracted to the corresponding data. The sensor also has some bad pixels that should be removed. They were detected using the white frame: pixels either “stuck” or exhibiting large variance over time were masked. Finally, any pixel value greater than 14’000 (out of 16’383) was masked, as this was observed to be the maximum value where the sensor was behaving linearly.

The reflectance was then computed. The sample and the white reference are non emissive in the wavelengths considered, and are considered lambertian. Therefore the following approximation can be used:

1 $${R}_{k}=\frac{{D}_{k}}{{W}_{k}}$$

No continuum removal was applied, as this may result in loss of information for spectra which don’t have sharp absorption features, and can be applied afterwards by the user of the dataset.

Finally, the HDR image was computed as the average of the non-masked values of the reflectance images:

2 $$.\text{hdr}.{\rm{h}}5[/\text{hdr}]=\frac{1}{n+1}\mathop{\sum }\limits_{k=0}^{n}\,\frac{{R}_{k}}{k}$$

Note that this is the average of multiple images of the same samples, in order to get one single 2D image and to decrease the amount of noise. No averaging between pixels was applied, as the spatial diversity has to be maintained. There are, in general, no outliers, as the bad pixels follow a fixed pattern, and therefore it is very unlikely that a bad measurement occurs on a otherwise valid pixel.

No further processing, like principal component analysis, was applied, as this would “smooth” the data captured, and therefore decrease the diversity used to train the neural network. This type of processing can, however, be applied independently by the users of the dataset, if required.

To isolate the sample from the background, a mask was constructed and applied to the data. The result was stored in.mhdr.h5[/hdr].

All the files contain also a /wavelengths array, which contains the list of band center, in nanometers.

### 3D reconstruction

This dataset contains 3D reconstructions of the samples as additional information. These 3D reconstructions can be useful to the user to understand specific aspects of the samples (for example, why a specific pixel has a different spectrum than the rest of the sample). The process was designed to be fully automated, and produced satisfactory results for most of the samples. For some of them, however, there are issues which create problems in the 3D reconstruction (such as holes, texture or shape artifacts). These issues were not corrected, since this data is not the main focus of the dataset.

Due to the rolling shutter of the webcam and the AC illumination, multiple frames had to be captured. Experience showed that 6 frames were sufficient to avoid any significant artifacts. The process employed is as follows:

1: **for** $\alpha \leftarrow 000,010,020\ldots 360$ **do**           ▹ Capture an image every 10 degrees

2:Angle of the sample ← *α*

3: **for** $k\leftarrow 1,2,\ldots 6$ **do**                     ▹ Capture 6 frames

4:    *I*_*k*_ ← webcam frame

5: **end for**

6: -im-α.jpg $$\leftarrow \frac{1}{6}{\sum }_{k=1}^{6}\,{I}_{k}$$

7: **end for**

8: **for** $\alpha \leftarrow 000,\,010,\,020\,\ldots \,360$ **do**             ▹ Compute smoothed image

9:*G*_*α*_ ← Gaussian filter (-im-α.jpg)

10: **end for**

For each *G*_*α*_, an image *M*_*α*_ is constructed by keeping only the pixels in the convex hull of the pixels which have changed between *G*_*α*_ and *G*_*α*+10_. This in effect removes the non-moving background behind the sample. The other pixels are replaced with black pixels.

The process is then the classical structure from motion 3D reconstruction^8^, with the exception that the features matching and the mapping are applied on *M*_*α*_ images. The other steps are performed with the -im-α.jpg images.

### Mineral characterization

Samples were characterized for their mineralogical class, crystallographic system, habit, luster, transparency, presence of cleavage, twinning and for their color. Table 1 includes the terms used to characterize the samples. Not all the terms are used in the current library, but they are still listed to indicate that they were considered while describing the sample, and to allow further extension of the library. They represent a compilation of commonly used terminology in mineralogy^7^, in view of future developments of the library. Additional information (i.e. chemical composition, provenance of the sample) are provided in the description.txt file. Mineralogical class and crystallographic system are well defined terms, as they relate to the internal order of crystals. Morphological crystallography (i.e. external form of minerals) incurs however some degree of subjectivity. Particularly for the description of the habit, different terms can be used to describe similar characteristics. In principle, the term euhedral was preferred when describing a crystal with well-formed faces but for minerals of the isometric system, the term cubes was preferred for habit description. At the same time, a mineral can be euhedral and prismatic when describing a crystal with four or more sides of similar length and width and being elongated in one direction. The term anhedral was used for mineral presenting no development of faces, microcristalline, massive or granular for aggregates of minerals with no visible macroscopic cristallographic characteristics, and amorphous for minerals lacking an internal atomic arrangement (i.e. opale). Habit terms such as lamellar, foliated, micaceous, bladed or tabular were used to describe mineral aggregate composed of scales or lamellae. The terms of fibrous, stellated, globular, botryoidal, reniform, mammillary, colloform apply to parallel or radiating groups of individual crystals, while the terms of acicular and filiform for minerals in isolated or distinct crystals. Luster and transparency definitions are described in^7^.

**Table 1:** Terms used for mineral classification. Other columns not present in this table are: cleavage, twinning and color.

Classification	System	Habit	Luster	Transparency
native element	monoclinic	euhedral	metallic	transparent
sulfides	triclinic	subhedral	non metallic	translucent
sulfosalts	hexagonal	anhedral	submetallic	non transparent
oxides	orthorombic	cubes	vitreous	opaque
hydroxides	isometric	microcristralline	pearly	
halides	tetragonal	massive	greasy	
carbonates		granular	silky	
nitrates		drusy	adamantine	
borates		lamellar	resinous	
phosphates		foliated	matte	
sulfates		micaceous		
tungstates		tabular		
silicates		bladed		
		fibrous		
		stellated		
		globular		
		botryoidal		
		reniform		
		mammillary		
		colloform		
		dendritic		
		reticulated		
		radiated		
		acicular		
		filiform		
		stalactitic		
		concentric		
		pisolitic		
		oölitic		
		banded		
		amygdaloidal		
		geode		
		concretion		
		prismatic		

## Data Records

The data is published on Zenodo as 3 different data records, depending on which hyperspectral data is included:

- ^9^contains the raw scan data
- ^10^contains the HDR data
- ^11^contains the masked HDR data
  Each data record is composed of a zip file for each sample, named *####*.zip, where *####* is the ID of the sample as available in Online-only Table 1. The data records also contain the ENVI template (provided in the file envi_template.xml.
  Each zip file contains the text description file: description.txt. It also contains the data of one or two measurement, A and possibly B. The files are named as follows (*####* is the sample ID, @ the measurement letter):
- *####-@.ply* and *####* -@.png: 3D reconstruction in Stanford PLY format, and associated texture file.
- @-im-000.jpg, @-im-010.jpg, …, @-im-360.jpg: JPEG images of the sample, the angle is in degrees. 0 degree correspond to the position used during scanning.
- @.scan.h5, @.hdr.h5, or @.mhdr.h5: hyperspectral data in HDF5 for^9,10^, and^11^ respectively.

## Technical Validation

**Fig. 2:**
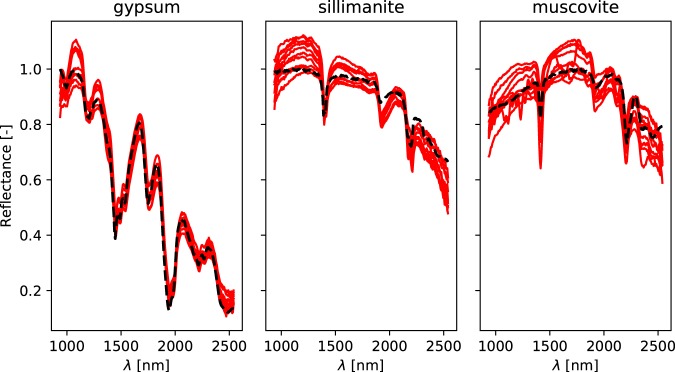
Comparison between spectra of the USGS spectral library (in black), and 10 out the 1’000 most similar spectra of the dataset (in red).

Comparing spectral libraries is challenging, as they consist of different samples, different sample preparation techniques and different data acquisition procedures. In general, the already existing libraries contain a single homogeneized measurement per sample, whereas the dataset presented in this library contains hundreds or thousands of datapoints. It should also be noted that these libraries also contain sometimes quite different spectra for a single mineral type. We observed, however, that the spectrum of these libraries can usually be found among the data points we measured, as it can be seen in Fig. 2.

Unfortunately, this approach is not sufficient for our dataset, since it contains many different data points for every mineral, and checking for inclusion of the spectra from established libraries (as in Fig. 2) would not show that these spectra are specific enough (in the worst case scenario: if all classes contain all the possible spectra, then inclusion would be satisfied but the library would be totally useless). It is therefore required to test that the different classes are different. This can be done using clustering algorithms. For this paper, we decided to use neural networks, as they are usually a efficient with a large amount of classes, and they are likely to be used by researchers using our dataset.

We therefore test that the measured spectra are specific to the sample class tested. To this mean, a common approach is to split the dataset, to have a classifier learn on one part of it, and to check the rate of correct classification on the part that was not used for learning. An efficient classification will indicate that the classes are well separated, while a bad classification accuracy will indicate that either the classes are not well separated, or the classifier was not efficient. A inefficient classifier could be due to either insufficient or incorrect training, or for it to be not able to capture the complexity of the data. Therefore, it is sufficient to show satisfactory classification efficiency for a given method (as the data size prevents a classifier to overperform by “luck”).

We followed this approach this approach by splitting randomly the hyperspectral dataset into three parts:

- The training dataset, containing 85% of the data points,
- The validation dataset, containing 10% of the data points,
- The test dataset, containing 5% of the data points.

The validation dataset is used to assess that the classifier is learning correctly, but the efficiency is checked on the test dataset after the learning is completed. Multiple different stuctures of artificial neural networks were trained in order to check how well the sample were identified.

The accuracy, while it depends of the classifier used, peaked at 98%. This is probably a limit due to the structure of the data. For example, it is possible that a few spectra of a sample are extremely similar to another class, and therefore the classifier cannot classify them correctly. The simplest one to achieve such correct classification rate is a multilayer perceptron of layer size 1024, 1024 and 256, with scaled input (by subtracting the mean and dividing by the standard deviation). The 98% of correct classification on the test dataset suggests that the dataset is of very good quality. Most of the 2% wrong classifications are on native elements and oxides samples, which mostly reflect light and don’t have well defined reflectance spectra.

## Usage Notes

To ease the re-use and the understanding of the image, a template is provided along the data files to load the data in ENVI. For.scan.h5 files, the template “fasnacht-hicsdata (SCAN)” should be used. For.hdr.h5 and.mhdr.h5 files, the template “fasnacht-hicsdata (HDR)” should be used. As ENVI doesn’t handle correctly matrices containing invalid values (NaN), they should be interpolated before using any algorithm. A step-by-step guide is provided on how to load the files in ENVI (Supplementary File 1).

We also provide hereafter the code to load a.h5 file in Python and MATLAB. These example are equivalent. We load the data and the wavelengths from the HDF5. The axis of the data should be transposed to be *y*, *x*, *λ*, as commonly used. NaN values in these matrix indicates masked information (due to saturation, bad pixels of the sensor, or mask).

- MATLAB code:
  data = permute(h5read(‘file.h5’, ‘/hdr’)[2, 1, 3);
  wavelengths = h5read(‘file.h5’, ‘/wavelengths’);
  imshow(nanmean(data, 3)’);
- Python code (requires numpy, h5py and matplotlib.pyplot):

d = h5py.File(‘file.h5’,‘r’)

data = numpy.transpose(d[‘/hdr’], (1, 2, 0))

wavelengths = d[‘/wavelengths’]

plt.imshow(numpy.nanmean(data), 2))

The 3D files can be loaded using CloudCompare Viewer (ccViewer), which can be downloaded here: https://www.danielgm.net/cc/.

### Supplementary information

Supplementary File 1

## Data Availability

The data acquisition and the hyperspectral data processing was performed using software developed inhouse. The software developed is available on GitHub: https://github.com/unine-chyn/hics. As the software setup for acquisition depends on the configuration and the hardware used, only an enumeration of the modules used is given. Three different classes of modules where used. Hardware related modules are specific to the hardware setup, and are likely to be modified with a different setup. Preview and control modules are used by the operator to control the camera and check that the process is performing satisfactorily. Finally, the plugins used for capturing data contain the algorithmic logic of the capture. Hardware related: hics.hardware.specimswir.camera (camera control), hics.hardware.specimswir.scanner (mirror scanner control), hics.hardware.specimswir.framegrabber (frame grabber), hics.hardware.ev3.ev3focus (LEGO focus control), hics.hardware.ev3.ev3rotater (LEGO rotating device), hics.hardware.webcam (webcam capture) Preview and control: hics.daemon.frameconverter (basic correction for preview), hics.gui (graphical user interface) Plugins for capturing data: hics.plugin.photogrammetry (capture data from the webcam), hics.plugin.record (record hyperspectral data), hics.plugin.autofocus (find the focus maximizing contrast), hics.plugin.labelprint (print the label for the sample), hics.plugin.autoscan (automate all steps required for the scan, using the various plugins) Once data is captured, the following commands were used (@ replaces the scan name): python3 –m hics.datafile.calibrate–input @.scan —output @.calibrated —max 14000 python3 –m hics.datafile.merge —input @.calibrated —output @.hdr —clean–required_images 2 # A mask should be generated by the user in @.mask.png python3 –m hics.datafile.maskdata —input @.hdr —mask @.mask.png —output @.mhdr. For the 3D reconstruction, after multiple experiments with various tools, it was found that the best output quality was obtained using COLMAP^8^ for the keypoints detection and OpenMVS^12^ for the construction of the pointcloud, the mesh, and the texturing. A small change was applied to COLMAP in order to orient the sample correctly. This change however doesn’t affect the quality of the result. OpenMVS was used without any modification. The code to automate the build of the tools and the processing of the images is available at https://github.com/unine-chyn/sfm-bundle.

## Online-only Table

**Online-only Table 1:** Summary of the contents of the dataset.

Mineral name	Sample IDs	Datapoints
Actinolite	0020, 0021, 0064	56840
Albite	0107	22521
Almandine	0025, 0026, 0073, 0074	17425
Andalusite	0014	18862
Anhydrite	0004	30832
Apatite	0089(2), 0090(2)	69062
Aragonite	0061	40111
Arsenopyrite	0087(2)	66333
Augite	0038	8503
Barite	0006	37130
Beryl	0075(2)	37743
Biotite	0049(2), 0050	45400
Blende	0086(2)	15596
Bronzite	0112	50452
Bytownite	0103	14666
Calcite	0010, 0011, 0052, 0078, 0079	112501
Cassiterite	0119, 0120	38535
Celestite	0000, 0001, 0002	56075
Chalcedony	0108(2), 0109(2)	96431
Chalcopyrite	0106	19375
Chlorite	0013	75802
Clinochlore	0126	45301
Coal	0081, 0082	44664
Copper	0101	14556
Diopside	0069	58959
Dolomite	0091(2), 0092(2)	76810
Enstatite	0047	31072
Epidote	0023, 0024	47306
Fluorite	0003(2), 0012(2)	98638
Galena	0053, 0054	17931
Garnet	0115	27331
Glaucophane	0016, 0017, 0076, 0077	200830
Goethite	0114	10717
Graphite	0083, 0084	20047
Grossular	0030, 0031	46592
Gypsum	0005(2), 0007, 0063	159829
Halite	0008, 0056	66201
Halloysite	0121, 0122	102028
Hematite	0039(2), 0085(2), 0095, 0096	165228
Hornblende	0046	9175
Hypersthene	0111	62932
Ilmenite	0116	16256
Kaolinite	0113	20764
Kyanite	0029	12320
Labradorite	0088(2), 0104(2)	62762
Limonite	0128, 0129	64550
Magnetite	0055, 0072	7103
Microcline	0071	60699
Montmorillonite	0123, 0124, 0125	87986
Muscovite	0034	62481
Nepheline	0097(2)	51930
Olivine	0065	7965
Omphacite	0019, 0067	108032
Opal	0102	34404
Orthoclase	0057	49824
Phlogopite	0045, 0070	105119
Pyrite	0042, 0048	31598
Pyrolusite	0117, 0118	45534
Pyrrhotite	0051	21572
Quartz	0009(2), 0035	126760
Rutile	0093	4959
Sanidine	0099(2)	49260
Serpentine	0018, 0068	78937
Siderite	0080	11651
Silicified wood	0127(2)	92935
Sillimanite	0032, 0033	70546
Sodalite	0043, 0060	61852
Sphalerite	0105	27485
Staurolite	0027, 0028, 0066	30628
Sulfur	0036, 0037	34751
Talc	0022, 0040, 0041, 0058, 0059	138317
Titanite	0094	4121
Tourmaline	0044, 0062	68419
Tremolite	0098(2), 0100	70913
Zircon	0110(2)	5110

## References

[1] Van der Meer FD (2012). Multi-and hyperspectral geologic remote sensing: A review. International Journal of Applied Earth Observation and Geoinformation.

[2] Kruse F (1993). The spectral image processing system (SIPS)-interactive visualization and analysis of imaging spectrometer data. Remote Sensing of Environment.

[3] Mountrakis Giorgos, Im Jungho, Ogole Caesar (2011). Support vector machines in remote sensing: A review. ISPRS Journal of Photogrammetry and Remote Sensing.

[4] Schneider Sven, Murphy Richard J., Melkumyan Arman (2014). Evaluating the performance of a new classifier – the GP-OAD: A comparison with existing methods for classifying rock type and mineralogy from hyperspectral imagery. ISPRS Journal of Photogrammetry and Remote Sensing.

[5] Krupnik Diana, Khan Shuhab (2019). Close-range, ground-based hyperspectral imaging for mining applications at various scales: Review and case studies. Earth-Science Reviews.

[6] Kokaly, R. F. *et al*. USGS spectral library version 7. Tech. Rep., US Geological Survey (2017).

[7] Klein, C. & Hurlbut, C. Jr. Manual of Mineralogy (after JD Dana), revised 21st edn (1999).

[8] Schönberger, J. L. & Frahm, J.-M. Structure-from-motion revisited. In *Conference on Computer Vision and Pattern Recognition (CVPR)* (2016).

[9] Fasnacht L, Vogt ML, Renard P, Brunner P (2018). Zenodo.

[10] Fasnacht L, Vogt ML, Renard P, Brunner P (2018). Zenodo.

[11] Fasnacht L, Vogt ML, Renard P, Brunner P (2018). Zenodo.

[12] Goldberg, A. V., Hed, S., Kaplan, H., Tarjan, R. E. & Werneck, R. F. Maximum flows by incremental breadth-first search. In Demetrescu, C. & Halldórsson, M. M. (eds) *Algorithms–ESA 2011*, 457–468 (Springer Berlin Heidelberg, Berlin, Heidelberg, 2011).

