# Supplementary material for: A 2D hyperspectral library of mineral reflectance, from 900 to 2500 nm
Source: Sci Data. 2019 Nov 11;6:268. doi: 10.1038/s41597-019-0261-9 (PMC6848079; doi:10.1038/s41597-019-0261-9)
Supplement: Supplementary file 1 [file 41597_2019_261_MOESM1_ESM.pdf]

Step-by-step guide on how to open the .h5 files with ENVI

1. Open the Data Manager (File -> Data Manager)

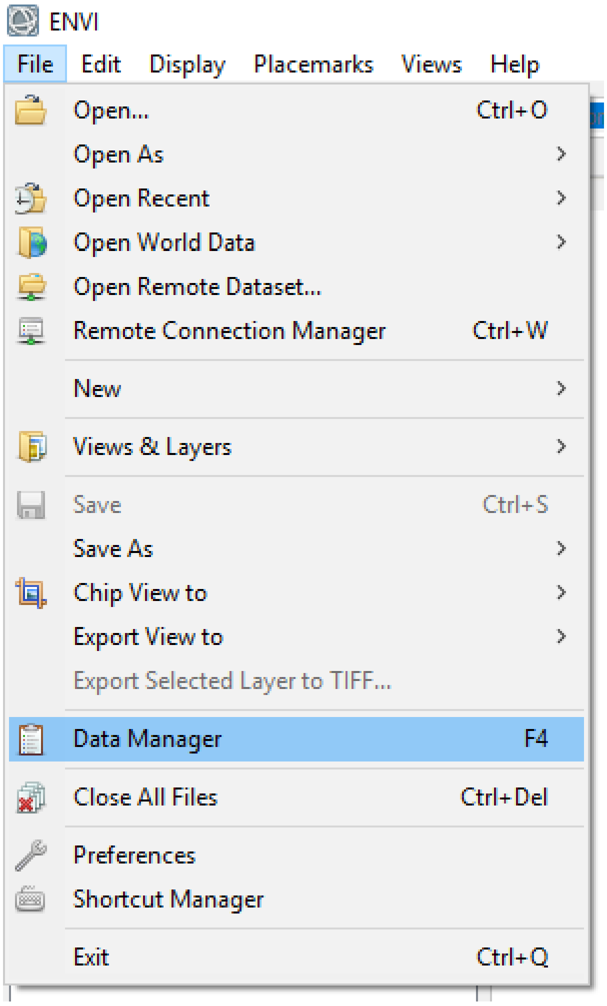

2. Open the .h5 file, using the Open button in the Data Manager. For this example, we will select a .hdr.h5 file, but the procedure is exactly the same for .scan.h5 or .mhdr.h5 files.

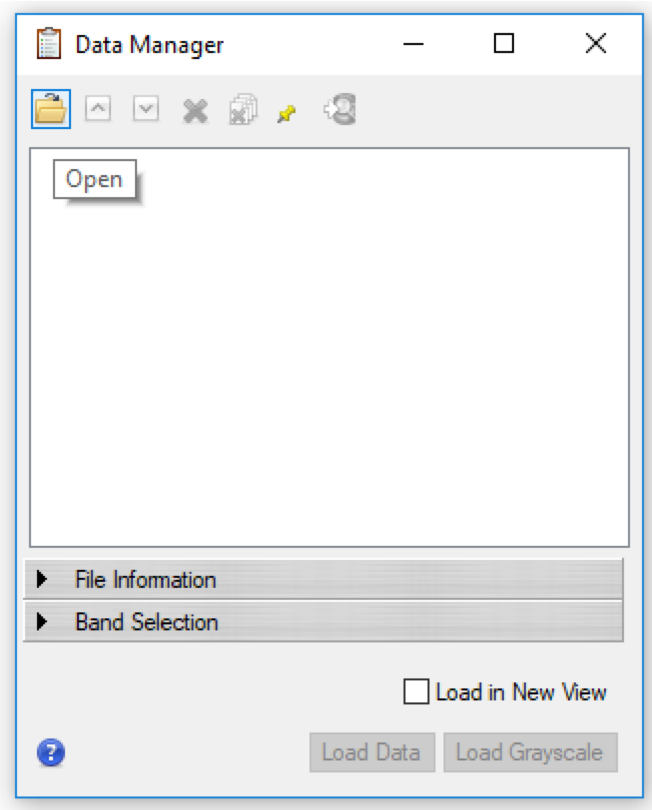

3. Select Templates -> Open Template, and select the provided .xml file. It is only needed to do this once per ENVI session.

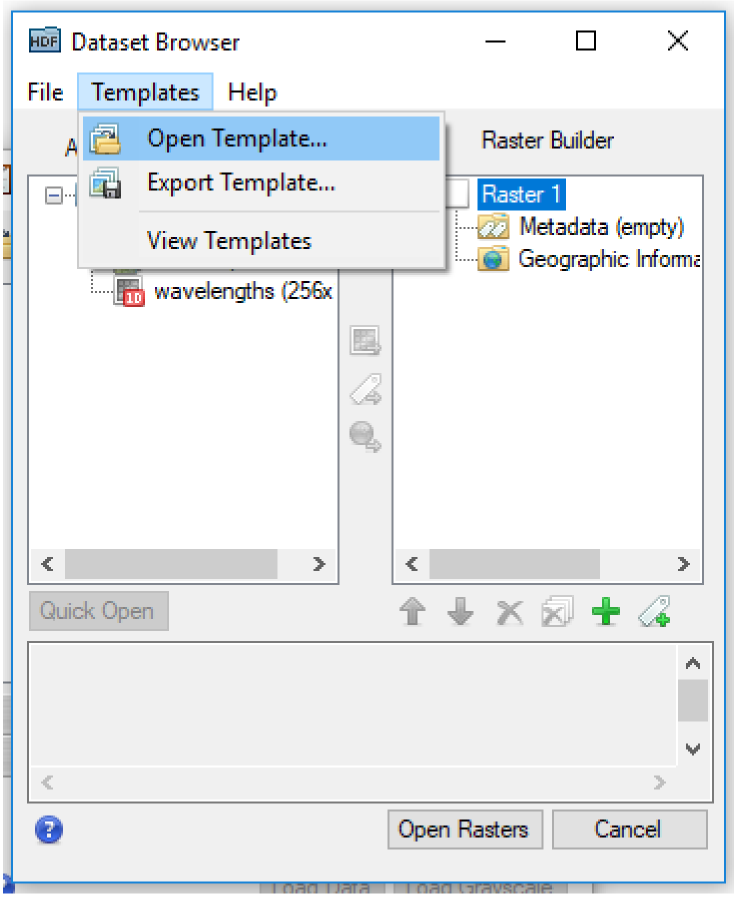

4. Apply the template using the template menu. For `.scan.h5` files, the template `fasnacht-hicsdata (SCAN)` should be used, and for `.hdr.h5` and `.mhdr.h5`, the template `fasnacht-hicsdata (SCAN)`

should be used.

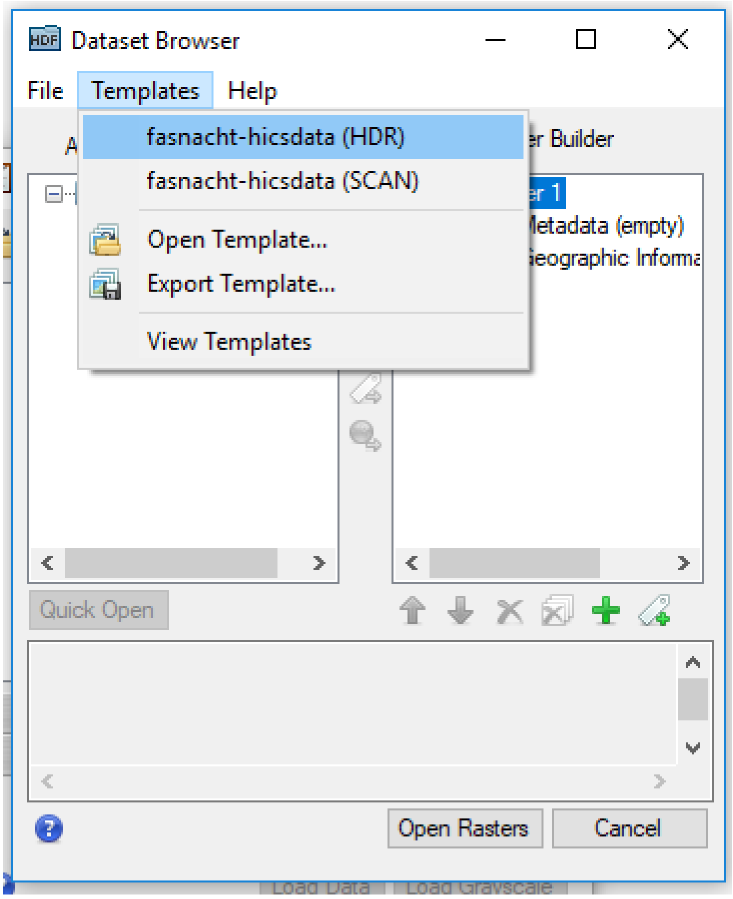

5. ENVI may complain that some of the data is missing. In that case, simply tick `Do not show this dialog again`, and press `OK`.

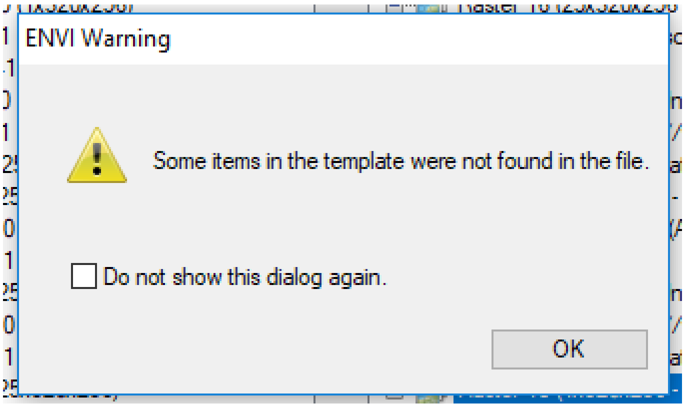

6. Once the template is applied, press `Open Rasters` to read and display the data.

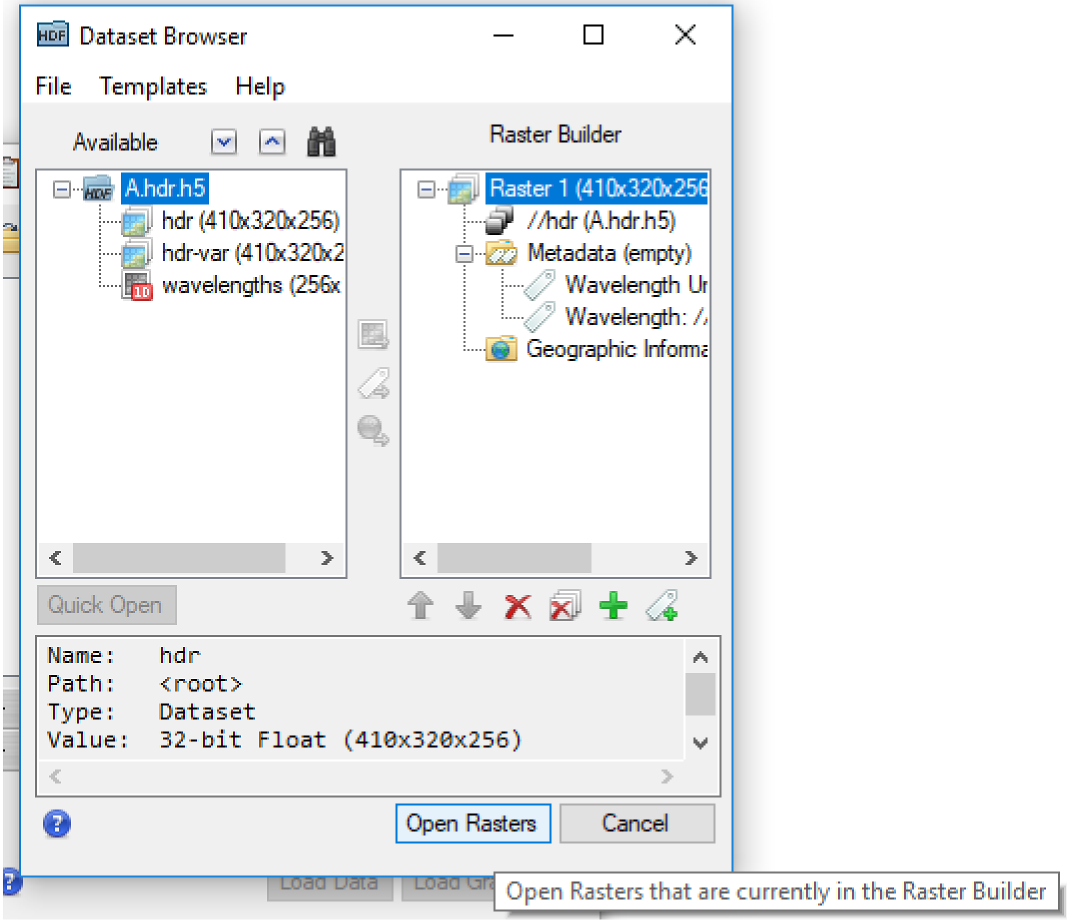

7. Here's an example of HDR data, with the spectral profile displayed for one point.

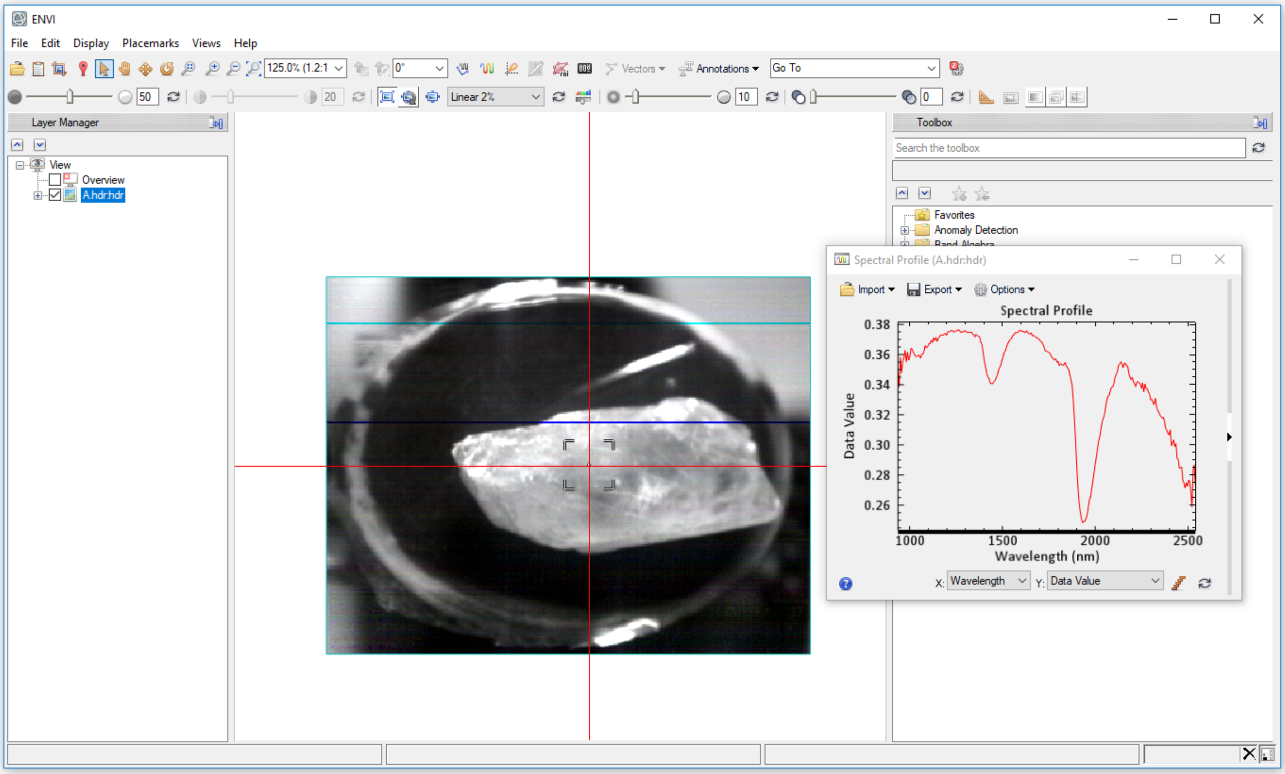

8. This is a similar image, but for the raw `.scan.h5` file. The horizontal lines are due to bad pixels of the sensor.

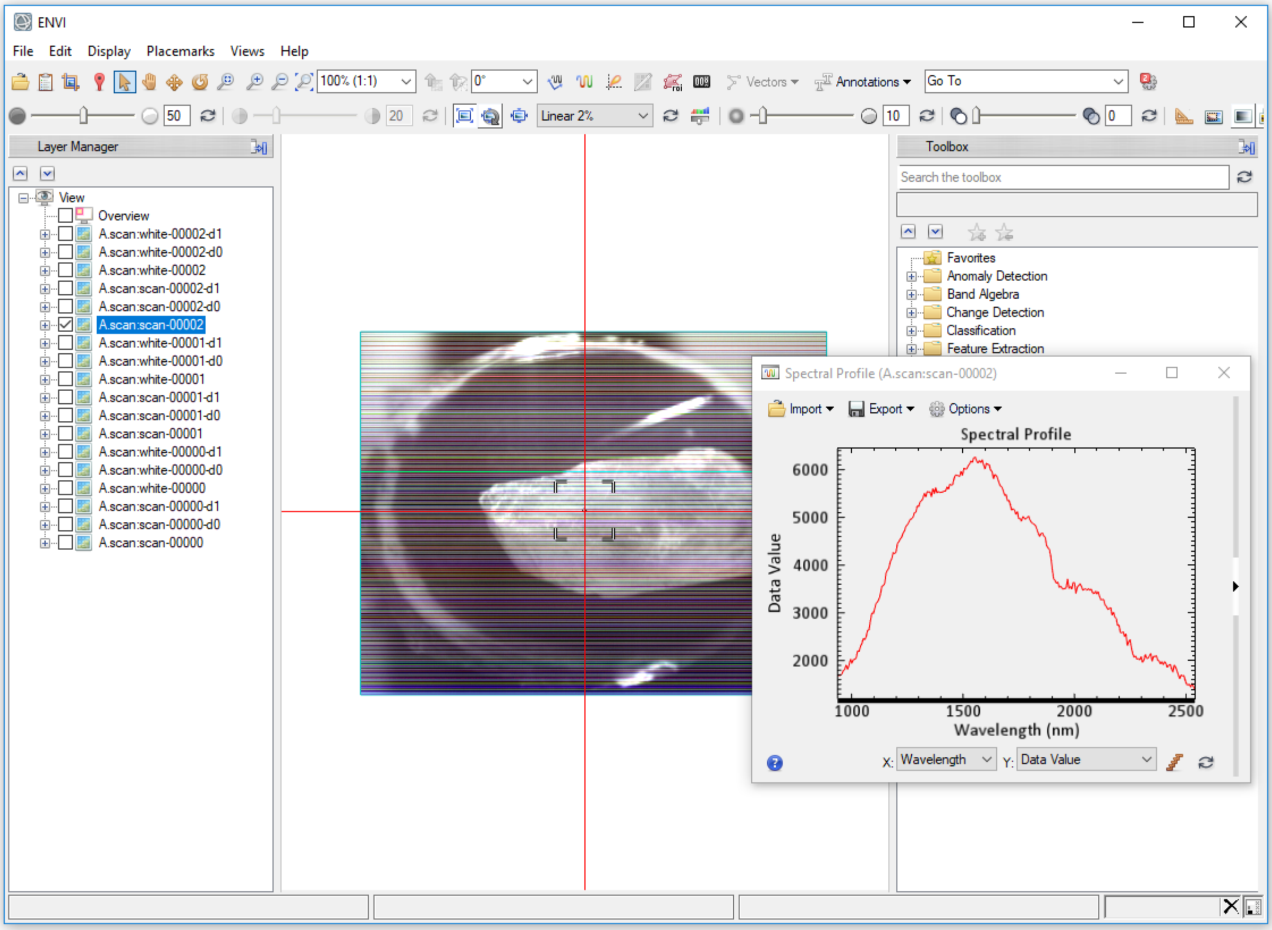
ENVI
File Edit Display Placemarks Views Help
100% (1:1)
Vectors
Annotations
Go To
Linear 2%
Layer Manager
View
Overview
A.scan:white-00002-d1
A.scan:white-00002-d0
A.scan:white-00002
A.scan:scan-00002-d1
A.scan:scan-00002-d0
A.scan:scan-00002
A.scan:white-00001-d1
A.scan:white-00001-d0
A.scan:white-00001
A.scan:scan-00001-d1
A.scan:scan-00001-d0
A.scan:scan-00001
A.scan:white-00000-d1
A.scan:white-00000-d0
A.scan:white-00000
A.scan:scan-00000-d1
A.scan:scan-00000-d0
A.scan:scan-00000
Toolbox
Search the toolbox
Favorites
Anomaly Detection
Band Algebra
Change Detection
Classification
Feature Extraction
Spectral Profile (A.scan:scan-00002)
Import
Export
Options
Spectral Profile
Data Value
6000
5000
4000
3000
2000
1000
1500
2000
2500
Wavelength (nm)
X: Wavelength
Y: Data Value
